# Bioethics for Medical Students Using a Novel Concept for Patient Workshops

**DOI:** 10.7759/cureus.96910

**Published:** 2025-11-15

**Authors:** Cristian Toma, Aida Petca, Ioana G Visan, Alexandra Munteanu, Viorel Jinga

**Affiliations:** 1 Department of Urology, Carol Davila University of Medicine and Pharmacy, Bucharest, ROU; 2 Department of Obstetrics and Gynaecology, Carol Davila University of Medicine and Pharmacy, Bucharest, ROU; 3 Department of Obstetrics and Gynaecology, Elias Emergency University Hospital, Bucharest, ROU; 4 Department of Medical Simulation, Carol Davila University of Medicine and Pharmacy, Bucharest, ROU; 5 Department of Urology, Clinical Hospital “Prof. Dr. Theodor Burghele”, Bucharest, ROU

**Keywords:** bioethics, education and training of medical students and doctors (specialist and phd)), medical education, medical simulation, standardized patient

## Abstract

Introduction

Standardized-patient (SP) encounters are widely used in medical education to enhance communication and professionalism. Although well established in residency training, their use in preclinical medical curricula - particularly in bioethics education - has been less explored.

Materials and methods

This single-institution pilot study involved six one-hour workshops conducted over three weeks at the Medical Simulation Department of the “Carol Davila” University of Medicine and Pharmacy, with 154 second-year medical students. Each session comprised two standardized-patient scenarios addressing emotionally complex bioethical situations, followed by structured debriefing. A five-point Likert-scale questionnaire, adapted from validated communication-training tools and reviewed by faculty experts for content validity, was used to collect students’ and lecturers’ perceptions. Data from 122 students and all three lecturers were analyzed using Kendall’s correlation, Cubist rule-based regression, and nonparametric ANOVA to identify associations among perception variables.

Results

Students rated communication, feedback, and session organization most positively. Perceived clinical competence was strongly associated with communication (τ = 0.79), SP feedback (τ = 0.68), and session structure (τ = 0.64). These three variables were also the most informative in the Cubist regression model (R² = 0.77). Nonparametric ANOVA confirmed significant associations between perceived competence and communication (p < .001), SP feedback (p < .001), and session organization (p = .037).

Conclusions

Students perceived SP workshops as engaging and valuable opportunities to apply ethical reasoning and communication skills in a realistic yet low-risk setting. As this study reflects self-reported perceptions from a single institution, its findings should be interpreted cautiously. Future multicenter and controlled studies combining perception and objective performance metrics are needed to determine the educational impact of SP-based bioethics training.

## Introduction

Up until the 1960s, medical students finished their studies without ever having an overseen conversation with a patient. Only recently have students started receiving guidance with learning to communicate [1]. In the last five decades, many medical schools implemented communication programs, and only in the last two decades these courses shifted from a didactic approach to an experiential learning method [1].

Bioethics, with concepts essential for all medical professionals during their careers, is taught in different ways across medical schools (lectures, written reflections or discussions in small or large settings) [2]. Some medical schools and departments of bioethics have developed programs enabling students through role-playing to apply theoretical knowledge with a student-centered approach [2].

Exposing medical students to the bioethical dimensions of medical practice offers advantages such as preserving the humanitarian tradition in medicine, aiding in the development of student identity, and clarifying power and responsibility [3]. Many times, preclinical medical students find it hard to fully understand how to use abstract concepts that are discussed in a theoretical manner and apply them during medical practice [4].

In a study conducted by Saber et al., results showed that simulated patients made the learning experience engaging and effective, aligning with previous research demonstrating their positive impact on moral competence. Learners showed stronger skills in recognizing moral issues than in ethical reasoning, decision-making, or executing moral actions, highlighting the need for targeted feedback in these areas [5].

The most beneficial ways of teaching or evaluating results of what has been taught in the field of bioethics are not straightforward [6]. Simulation has the advantage of offering students the possibility of deliberate practice in a realistic environment, being guided in the process, and receiving feedback [4].

When it comes to teaching communication skills, students incline towards experiential methods, such as interviewing standardized patients (SPs) or real patients [7]. Practicing patient contact and communication early on with SPs gives students the chance to exercise clinical judgement, allowing them much-needed opportunities for repetition. It appears that students who participate in practice runs with standardized patients feel more prepared for future standardized patient encounters that are graded [8]. Many studies support the idea that students who follow medical schools that utilize standardized patients as educational tools feel more confident, less anxious, and have better communication skills [9].

Given that bioethics education often addresses complex, emotionally charged, and ethically nuanced clinical scenarios, standardized patient encounters provide a unique opportunity for students to actively engage in realistic interactions that foster empathy, communication, and ethical reasoning skills that are difficult to cultivate through traditional lectures alone.

Our study was developed with the intention of introducing novice students to simulation-based practical sessions (through guided standardized patient encounters) and assessing their opinion regarding the impact and perceived advantages of simulated sessions with standardized patients.

## Materials and methods

Study design and duration

This pilot study was conducted over a three-week period (15th of November 2024 - 29th of November 2024) at the Medical Simulation Department of the "Carol Davila" University of Medicine and Pharmacy in Bucharest, Romania*.*

Ethical approval and informed consent

Ethical approval for this study was obtained from the Ethics Committee of the University (approval no. 17465/28.06.2024). All students signed an informed consent prior to participating in these sessions.

Workshop design and objectives

We designed and implemented six one-hour workshops using standardized-patient encounters to expose second-year medical students to structured ethical communication scenarios and to evaluate their perceptions of the experience rather than objective skill acquisition of these medical students enrolled in the Bioethics and Academic Integrity course.

Workshop structure and scenarios

The workshops were offered as voluntary supplementary sessions within the Bioethics and Academic Integrity course. Attendance did not influence academic grading. No randomization or mandatory participation was applied, and students who consented were included consecutively until reaching the planned capacity for each session. Within each group, one student volunteered to conduct the simulated interview, while peers served as observers instructed to document both strengths and areas for improvement using a structured checklist to reduce social desirability bias.

Each workshop was structured around two clinical scenarios, followed by a debriefing session. The format of each session included: 20 minutes for the first SP encounter, 20 minutes for the second case, and 20 minutes dedicated to group reflection and feedback.

Case content and curriculum integration

The scenarios were developed in alignment with the theoretical content of the bioethics curriculum and addressed emotionally complex patient interactions - a young woman presenting with generalized anxiety disorder after a traumatic experience, and a caregiver supporting a terminal lung cancer patient.

Participant preparation and roles

Prior to participation, students received an informative email detailing the session structure, learning objectives, and individual roles. Each workshop included approximately 25 participants. One volunteer conducted the medical interview, while the rest observed and documented both positive behaviors and areas for improvement.

Post-encounter debriefing and feedback

All participants engaged in post-encounter discussions and received feedback from both the standardized patient and the session facilitator.

Standardized patient training

**Figure 1:**
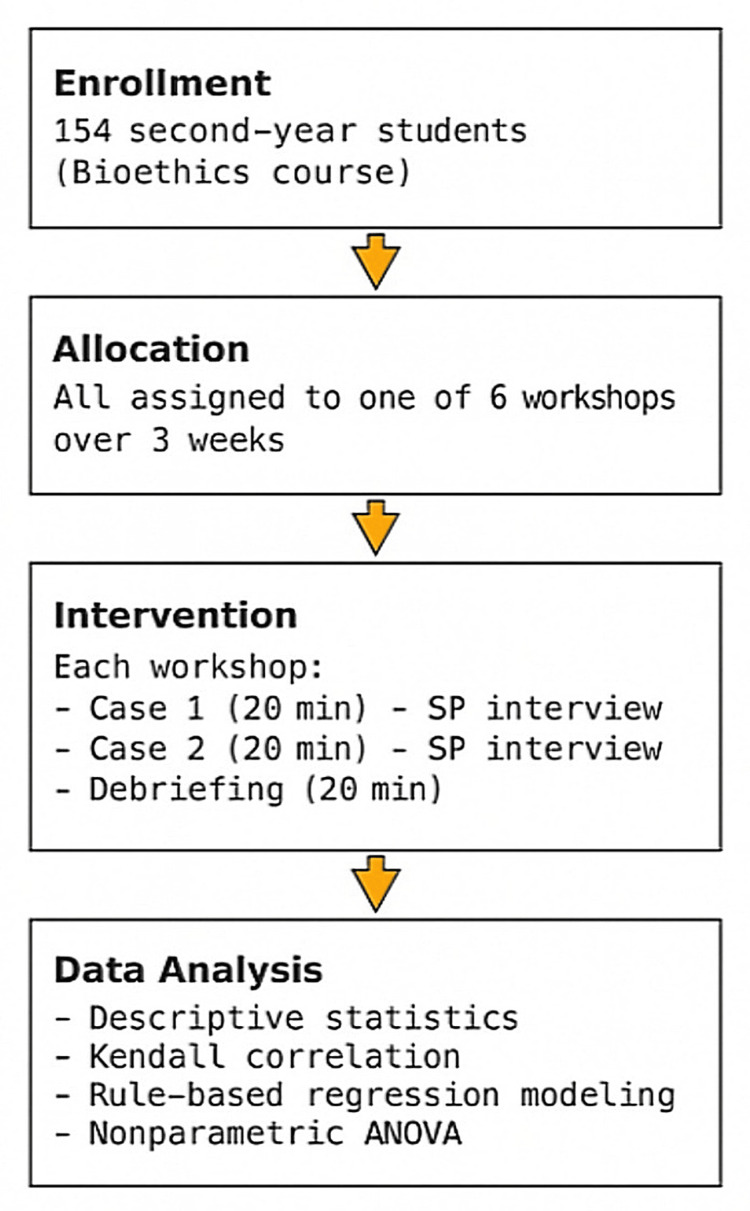
Study design

The standardized patients were portrayed by four senior medical students (fourth- and fifth-year) who had volunteered and undergone targeted training. As seen in Figure 1, the training consisted of four two-hour sessions held weekly over a month, with scripted case rehearsals, peer feedback, and faculty supervision to ensure consistency and realism across all workshops.

Participant demographics and feedback evaluation

A total of 154 second-year students participated, all receiving identical exposure to the two cases. At the end of each session, students and lecturers completed a Likert-scale feedback questionnaire that was specifically created for this workshop (inspired and adapted after Pinar et al. [10]), evaluating multiple domains: session organization, appropriateness of the location, SP preparedness, clarity of learning objectives, adequacy of allocated time, perceived improvement in communication and clinical skills, usefulness of feedback, emotional response to the experience (e.g., stress), and perceived value of SP-based education in the broader medical curriculum (1 = strongly disagree to 5 = strongly agree). The flow diagram describing the process can be seen below in Figure 1.

Data analysis

We aimed to identify which student-rated variables were most strongly associated with perceived clinical competence. To explore these associations, we used both statistical and machine-learning approaches. The Aligned Rank Transform (ART) method was applied to perform nonparametric ANOVA on Likert-scale data [11,12]. All questionnaire responses were numerically coded from 1 (strongly disagree) to 5 (strongly agree) and imported into R statistical software (R Foundation for Statistical Computing, Vienna, Austria) for preprocessing. Data were screened for completeness, with missing responses (<5%) excluded pairwise and no imputation applied.

We then calculated Kendall’s correlation and adjusted mutual information for each variable pair. The former was chosen as a robust nonparametric alternative to Spearman’s correlation, while the latter quantifies nonlinear associations (values range from 0 = no association to 1 = maximum association). Finally, predictors of perceived clinical competence were explored using a Cubist rule-based multivariate regression model [13]. All analyses were conducted in R, with a significance threshold of p < 0.05. The analysis was primarily exploratory, intended to identify internal associations among perception variables rather than to establish predictive validity or causality.

## Results

Out of the 154 second-year medical students enrolled in the workshops, 122 (79.2%) completed the post-session feedback questionnaire. Additionally, all three participating lecturers submitted the evaluation form addressed to faculty.

Demographic characteristics of the students were: 66 (54%) identified as male and 56 (46%) as female. Most participants were 20 years old (n=37, 30%), followed by 21-year-olds (n=31, 25.4%), 19-year-olds (n=30, 24.6%), and smaller proportions between 18 and 29 years.

When asked whether this was their first encounter with a SP out of 122 respondents, 105 (86.1%) students responded affirmatively.

Perceptions of workshop structure and organization

Session organization was highly rated: out of 122 participants, 74.6% of students (n=91) strongly agreed the workshop was well organized, 19.7% agreed (n=24), while only 5.7% remained neutral (n=7).

Workshop location was perceived as very appropriate by 75 students (61.5% strongly agree), with an additional 19 (15.6%) participants agreeing. Neutral responses were given by 17 (13.9%), while 11 (9%) disagreed or strongly disagreed.

SP preparation was positively evaluated by most respondents: 91 (74.6%) strongly agreed the SP was well prepared, 18 (14.8%) agreed, and 14 (10.7%) were neutral.

Clarity of learning objectives was confirmed by 113 (92.6%) of students, with only 3 (2.4%) expressing disagreement.

Perceived impact on learning and skills

**Figure 2:**
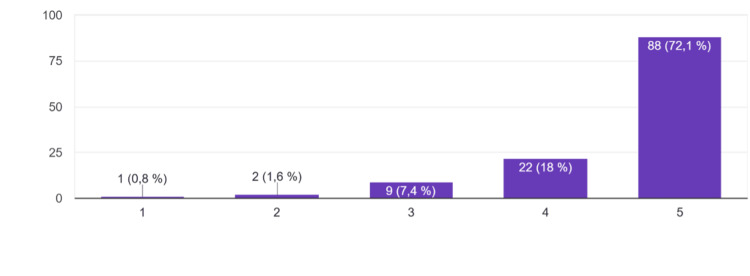
Perceived communication skills development -"I believe the session helped me improve my communication skills" Responses were recorded on a 5-point Likert scale: 1 = Strongly disagree, 2 = Disagree, 3 = Neutral, 4 = Agree, 5 = Strongly agree.

When it came to perceived communication skill development, as seen in Figure 2, 88 participants (72.1%) strongly agreed the session was helpful, and 22 (18%) agreed, with 3 (2.4%) indicating disagreement.

**Figure 3:**
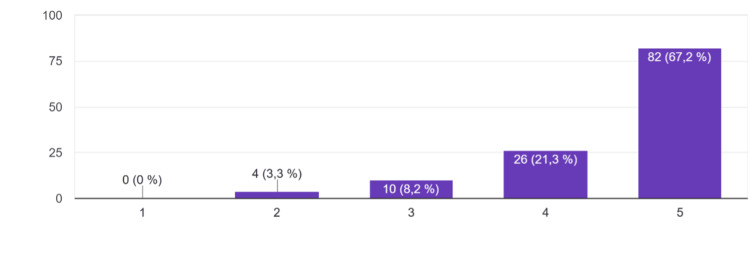
Perceived Competence development -"I believe the session helped me improve my clinical competence." Responses were recorded on a 5-point Likert scale: 1 = Strongly disagree, 2 = Disagree, 3 = Neutral, 4 = Agree, 5 = Strongly agree

In terms of perceived improvement in clinical competence, as shown in Figure 3, 82 students (67.2%) strongly agreed and 26 (21.3%) agreed, while 10 (8.2%) remained neutral and 4 (3.3%) disagreed.

Feedback quality and educational value

**Figure 4:**
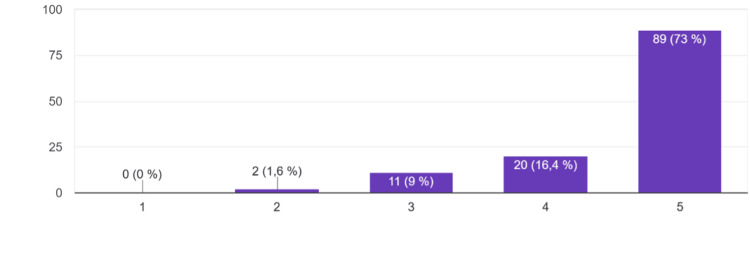
"I found SP's feedback very helpful" Responses were recorded on a 5-point Likert scale: 1 = Strongly disagree, 2 = Disagree, 3 = Neutral, 4 = Agree, 5 = Strongly agree.

Feedback from the standardized patients was rated as very helpful, as shown in Figure 4, by 89 participants (73%), with an additional 20 (16.4%) agreeing.

Similarly, lecturer feedback was perceived as very helpful by 73% of students (n=89), with 14.8% (n=18) agreeing and only 3.3% (n=4) expressing disagreement.

When asked about the educational value of standardized patient encounters, 88 (72.1%) students strongly agreed they would benefit from more such experiences, 21 (17.2%) agreed, and only one respondent strongly disagreed.

Emotional response to the simulation

Regarding emotional impact, 61 (50%) students strongly agreed that the experience was stressful, 11 (9%) agreed, and 19 respondents (15.6%) were neutral. A total of 31 participants (25.5% strongly disagreed or disagreed) reported that the experience was not stressful.

Faculty perceptions

All three participating lecturers reported this was their first time engaging in a simulated session using SPs. All faculty members strongly agreed that the session was well organized, that the location was appropriate, and that the SPs were well prepared. They all confirmed that the activity helped students improve both communication and clinical competence and supported the inclusion of more SP-based sessions in medical training.

Statistical analysis

**Figure 5:**
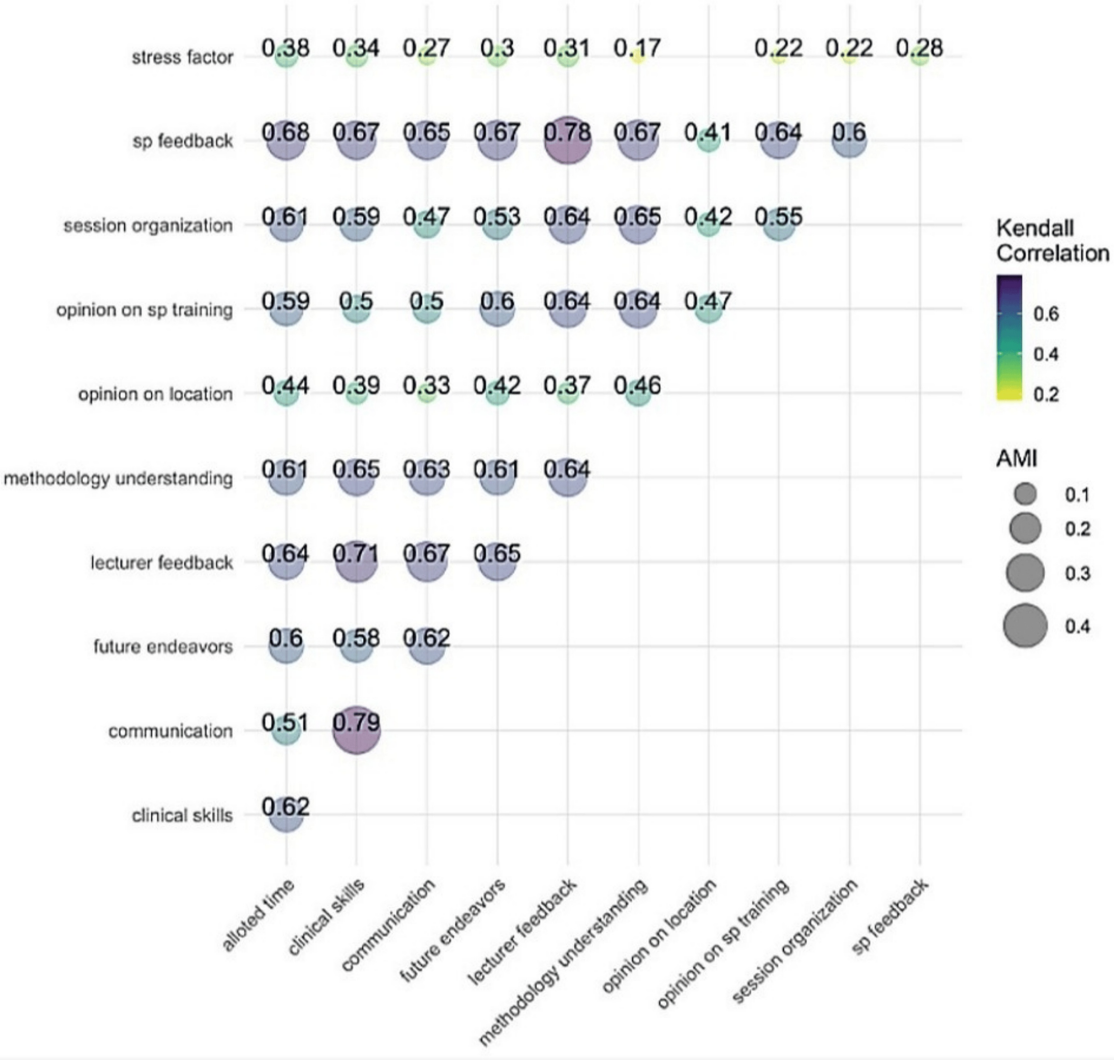
Kendall Correlation analysis

The Kendall correlation analysis showed a strong positive association between students' perceived improvement in clinical competence and key components, including communication skills (τ = 0.79), SP feedback (τ = 0.67), and session organization (τ = 0.59). Feedback from lecturers and understanding of the session methodology also showed substantial positive correlations with both perceived communication and clinical skill outcomes as seen in Figure 5.

To explore the predictive factors, a rule-based regression model was used. The model achieved an R2 of 0.77, identifying communication, SP feedback, and session organization as the most relevant contributors to student’s perceived clinical competence. The model segmented students into three distinct decision-rule groups based on communication scores and perceived time adequacy.

SP feedback (τ = 0.78) demonstrates the highest correlation with lecturer feedback, indicating that students perceived both feedback sources as complementary and equally valuable for learning. However, communication, SP feedback, and lecturer feedback emerged as the most influential factors enhancing students’ perceived clinical skill development, while factors like stress or location showed weaker associations (τ < 0.4).

ART was conducted to assess the statistical significance of these associations. Results are presented in Table 1.

**Table 1:** Aligned Rank Transform ANOVA – Predictors of Perceived Clinical Skills

Covariate	F value	P value
Communication	48	< .001
SP Feedback	14.6	< 001
Session Organization	4.6	0.04
Allotted Time	0.7	0.42

## Discussion

Overall, the first standardized patient session organized for second-year medical students attending classes for the Bioethics and Academic Integrity Discipline was well received by all lecturers and highly regarded by most students who completed the survey.

The findings of this study suggest that perceived communication skills, feedback quality, and session organization are the most influential factors in shaping second-year medical students' confidence in their clinical competence following SP workshops. The robust correlations observed in the Kendall analysis were further supported by both the Cubist machine learning model and nonparametric ANOVA analysis.

Students who rated their communication more positively also tended to report higher perceived clinical competence, suggesting that these perceptions are closely interrelated. The perceived value of SP feedback further highlights the importance of structured reflection and debriefing in shaping students’ learning experience. These findings reflect subjective associations among perception variables rather than objective measures of competence.

It is interesting that while students often highlight time limitation in simulated environments, the attributed time was not statistically significant in predicting perceived clinical skills, suggesting that students’ perception of competence is more tightly linked to interaction quality rather than duration.

These results emphasize the perceived importance of well-prepared standardized patients and constructive feedback, particularly in preclinical years when direct patient contact is limited. The consistent patterns observed across statistical analyses suggest internal coherence within the data and point to the potential value of early SP-based learning in medical curricula.

Various approaches have been explored in teaching bioethics, from traditional lectures to more interactive and simulation-based formats. In a quasi-experimental study with 33 nursing students, a bioethics course focused on empathy and dignified death led to greater awareness and self-reported empathy after students engaged in communication activities following classroom instruction [14].

Traditional lectures continue to have value, particularly for introducing basic ethical concepts to large groups. However, several authors emphasize that active learning methods encourage deeper understanding and reflection. Khorasani and Ebrahimi compared lecture-based instruction with video-taped standardized-patient cases when teaching informed consent. They found that the lecture format remained effective, especially when combined with SP-based scenarios [15]. Similarly, Vedavathi et al. noted that both students and physicians preferred discussion-based learning to lectures or PowerPoint presentations, as ethical concepts are abstract and often require dialogue for genuine comprehension [16].

Interactive methods such as role-playing and patient narratives have also shown promise. D’Souza et al. observed that when students had the chance to take on the physician’s role in interprofessional role-plays, they reported improved communication and teamwork, along with stronger empathy toward patients [17]. Comparable findings were reported in studies where expert-guided interactions with simulated patients enhanced ethical reasoning and reflective learning, further highlighting the perceived educational benefit of SP sessions [18].

Recent work suggests that virtual standardized patients can offer similar advantages. One study comparing live and virtual SP encounters found equivalent improvements in diagnostic reasoning and communication, suggesting that virtual simulations can broaden access to experiential bioethics training when live encounters are impractical [19].

Even with these encouraging results, the literature still lacks clear guidelines for evaluating the effectiveness of different bioethics teaching methods, and reported outcomes vary considerably [20]. Small-group teaching formats are often preferred because they promote discussion, self-reflection, and transformative learning, though additional research is needed to confirm their broader applicability [21].

Overall, the use of standardized patients (whether live or virtual) can create a safe environment for learners to apply ethical principles, strengthen communication, and receive immediate feedback [22]. Within this context, our pilot study adds to current evidence by exploring how both students and tutors perceive early SP-based workshops in bioethics education. For many participants, this represented their first experience interacting with a patient, even a simulated one, offering valuable insight into how such encounters are received and interpreted during the preclinical stage of training.

Limitations and future directions

This study has several limitations that should be acknowledged. The sample size was relatively small, particularly regarding faculty participants, and the student cohort was limited to second-year medical students. Because of this, the findings may not reflect perceptions across different training levels or institutions.

The study was conducted at a single university and used a single cohort, which limits generalizability to other educational and cultural settings. Moreover, participation was voluntary, which may have introduced self-selection bias, as students interested in communication or ethics may have been more motivated to engage positively with the workshop experience.

Another methodological limitation is the absence of a control or comparison group, which precludes any causal interpretation of the findings. The outcomes represent subjective perceptions, collected immediately after participation, and do not measure objective competence or knowledge acquisition. Post-session enthusiasm, novelty effects, and social desirability bias may also have influenced students’ responses.

The questionnaire, while adapted from validated communication-simulation tools and reviewed by faculty for content validity, was not psychometrically validated for factor structure, internal consistency, or construct validity in this specific context. Similarly, the portrayal of standardized patients by senior medical students, rather than professional actors, may have introduced variability in case fidelity and response standardization.

The study design did not include longitudinal follow-up; therefore, we cannot determine whether perceived benefits persisted over time or translated into actual performance improvements. Future studies should combine perception measures with objective assessments, such as OSCE performance, expert ratings, or validated communication-skill scales, and should ideally use controlled or comparative designs to determine educational effectiveness.

Despite these limitations, this exploratory pilot provides meaningful insight into how preclinical students and faculty perceive standardized-patient workshops within bioethics education. The findings highlight feasibility, acceptance, and perceived value, laying the groundwork for future mixed-methods and multicenter studies aimed at refining early SP-based curricula.

## Conclusions

In this exploratory pilot study, preclinical students perceived standardized-patient workshops as engaging and relevant learning experiences that improved their confidence in communication and ethical reasoning. The sessions were valued for their realism, feedback, and supportive environment. However, as data were based solely on post-session self-reported perceptions without objective or comparative measures, no conclusions can be drawn regarding actual skill improvement. These findings highlight the perceived feasibility and educational value of integrating standardized-patient encounters into early bioethics training.
